# Assessment and management of disorders of gut–brain interaction in patients with eating disorders

**DOI:** 10.1186/s40337-022-00731-6

**Published:** 2023-02-13

**Authors:** Micaela Atkins, Helen Burton Murray, Kyle Staller

**Affiliations:** 1 Division of Pediatric Gastroenterology, Hepatology and Nutrition, Department of Pediatrics, Massachusetts General Hospital, 55 Fruit Street, Boston, MA 02114 USA; 2 Division of Gastroenterology, Center for Neurointestinal Health, Massachusetts General Hospital, 55 Fruit Street, Wang 5, Boston, MA 02114 USA; 3 Department of Psychiatry, Harvard Medical School, 401 Park Drive, Boston, MA 02215 USA; 4 Department of Medicine, Harvard Medical School, 25 Shattuck St, Boston, MA 02115 USA

**Keywords:** Disorders of gut–brain interaction, Functional gastrointestinal disorders, Feeding and eating disorders, Anorexia nervosa, Bulimia nervosa, Avoidant/restrictive food intake disorder, Binge-eating disorder, Irritable bowel syndrome, Dysphagia, Dyspepsia, Constipation

## Abstract

Disorders of gut–brain interaction (DBGI), also known as functional gastrointestinal disorders, are common in individuals with eating disorders, and may precede or perpetuate disordered eating. Understanding the pathophysiology of common gastrointestinal symptoms in DGBI can be important for the care of many patients with eating disorders. In this review, we summarize the literature to date on the complex relationship between DBGI and eating disorders and provide guidance on the assessment and management of the most common symptoms of DBGI by anatomic region: esophageal symptoms (globus and functional dysphagia), gastroduodenal symptoms (functional dyspepsia and nausea), and bowel symptoms (abdominal pain, bloating and constipation).

## Plain English Summary

Disorders of gut–brain interaction, also known as functional gastrointestinal disorders, are common in individuals with eating disorders and can cause symptoms that affect all parts of the gastrointestinal system. In this review, we describe common symptoms of disorders of gut–brain interaction and recommendations for their assessment and management.

## Introduction: disorders of gut–brain interaction and eating disorders

Disorders of gut–brain interaction, (DGBI; also known as functional gastrointestinal (GI) disorders) are among the most common GI diagnoses, resulting in significant morbidity worldwide [1]. These disorders are classified based on chronic GI symptoms which occur in the absence of inflammation or structural abnormalities (i.e. there is no underlying cancer, ulcer etc.) [2]. Patients with DGBI often have eating-related concerns, and there is growing recognition of the relevance of DGBI symptoms to eating disorders (EDs). Both EDs and DGBIs lie at the interface of the brain-gut connection, with input from biological, psychological, and social factors [3, 4].

While many providers are familiar with the organic GI conditions which may occur in the setting of EDs, such as acute gastric dilation, elevated liver enzymes or superior mesenteric artery syndrome, the complex interplay between EDs and DGBI may be overlooked. An understanding of DGBI symptoms, diagnosis and management is therefore important for ED providers, who need to develop a measured approach to testing and treating GI symptoms in their patients.

### What are DGBIs?

DGBIs are a group of disorders comprised of chronic GI-related symptoms, such as abdominal pain, nausea, bloating, constipation and diarrhea, in the absence of structural or inflammatory disease. The pathophysiology of DGBI remains incompletely understood, and involves alterations in physiologic factors such as GI tract motility, immune function, pain sensation and autonomic function, as well as psychosocial factors [2, 5]. Key to this process is the gut–brain axis, which mediates signaling between the enteric nervous system of the GI tract and the central nervous system (see Table 1).

**Table 1:** Key Terms Related to DGBI Symptoms

Term	Definition
Brain-Gut axis	Bidirectional neurohumoral communication between the gastrointestinal tract and central nervous system
Brain-Gut behavior therapy	Evidence-based psychotherapeutic interventions that target brain-gut interaction, such as relaxation training, mindfulness training, cognitive behavioral therapy for GI disorders, and gut-directed hypnotherapy
Complementary therapy	Natural products or mind–body medicine which are used in addition to or as an alternative to standard medical treatments
Enteric nervous system	A complex network of nerves which line the GI tract and controls GI tract motility and function
Manometry study	Study which measures pressures and patterns of GI organ motility and sensation
Motility	Movement of contents through the GI tract, mediated by complex signaling between nerves and muscles
Organic	In the context of DGBI, used to describe possible underlying anatomic (structural) or inflammation-related causes of symptoms (e.g. cancer, ulcer causing abdominal pain)
Prokinetic	Pharmacological agents which promote movement of contents in the GI tract
Neuromodulators	Pharmacological agents which primarily have activity in the noradrenergic, serotonergic, dopaminergic systems in the brain-gut axis
Visceral hypersensitivity	A key pathophysiological mechanism involved in pain sensation in DGBI, causing heightened perception of gut stimuli

DGBIs are diagnosed with the Rome criteria, which classifies DGBI based on symptoms within anatomic regions (e.g. esophageal, gastroduodenal, bowel) [2]. The most recent schema, Rome IV, published in 2016, comprises 33 adult and 20 pediatric DGBIs, comprehensive review of which is outside of the scope of this review. Thus, in this review, we focused on the DGBI symptoms and disorders which present most frequently in patients with EDs (see Table 2).

**Table 2:** Disorders of gut–brain interaction most commonly seen in patients with EDs by anatomic region

Esophageal disorders	Gastroduodenal disorders	Bowel disorders
Globus Functional dysphagia	Functional dyspepsia Nausea and vomiting disorders	Irritable bowel syndrome Functional constipation Functional abdominal bloating/distension

Management of DGBI involves a multimodal approach with pharmacologic and brain-gut behavior therapies and specialist care as needed (e.g. pelvic floor physical therapy). Dietary therapies (e.g. the low FODMAP diet) and complementary/alternative therapies (e.g. acupuncture) are often recommended, with some dietary therapies having substantial evidence for certain DGBIs [6] and growing interest and study of complementary/alternative therapies [7]. Due to the contraindication of restrictive dietary therapies for individuals with EDs and the limited research on complementary/alternative therapies, we focus on pharmacologic and brain-gut behavior therapies in this review. In addition to standard management of GI symptoms with peripherally acting agents such as proton-pump inhibitors and laxatives, pharmacologic treatment of DBGI involves neuromodulators, which act on the gut–brain axis [8]. Neuromodulators, such as tricyclic antidepressants (TCAs) and selective serotonin reuptake inhibitors (SSRIs), have effects on GI sensorimotor function and may treat comorbid psychiatric symptoms [9] (see Table 3). Brain-gut behavior therapies target GI symptoms and include a variety of cognitive-behavioral therapies, gut-directed clinical hypnosis, mindfulness-based interventions, and psychodynamic-interpersonal therapies [10, 11]. Brain-gut behavior therapies are potentially promising for individuals with EDs, and have been proposed to target DBGI symptoms in EDs [12].

**Table 3:** Commonly used medications for DGBI symptoms

Name	Drug class	Dose	Therapeutic use	Adverse effects
Secretagogues				
Linaclotide	Guanylate Cyclase-C Agonist	72–290 mcg QD	Constipation, abdominal pain	Diarrhea, abdominal distension, abdominal pain
Lubiprostone	Chloride channel activator	8–24 mcg BID	Constipation, abdominal pain	Nausea, diarrhea, headache
Plecanatide	Guanylate Cyclase-C Agonist	3 mg QD	Constipation, abdominal pain	Diarrhea, abdominal distension
Tenapanor	Sodium-hydrogen exchanger inhibitor	50 mg BID	Constipation, abdominal pain	Diarrhea, abdominal distension, dizziness
Prokinetic Agents				
Azithromycin	Motilin receptor agonist	250–500 mg QD	Dyspepsia, gastroparesis	Tachyphylaxis, QT prolongation, abdominal pain
Erythromycin	Motilin receptor agonist	50 mg qAC and qHS	Dyspepsia, gastroparesis	Tachyphylaxis, QT prolongation, abdominal pain
Domperidone	Dopamine receptor antagonist	10 mg TID	Dyspepsia, gastroparesis	QT prolongation, arrhythmia, galactorrhea
Metoclopramide	Dopamine receptor antagonist	2.5–10 mg qAC and qHS	Dyspepsia, nausea, gastroparesis—good for acute, self-limited symptoms	QT prolongation, Eextrapyramidal symptoms, akathisia, galactorrhea
Prucalopride	5HT-4 Agonist	2 mg QD	Constipation, dyspepsia, nausea	Abdominal pain, headache, diarrhea
Neuromodulatory Agents				
Buspirone	5HT1-agonist	15– 30 mg BID	Dyspepsia, nausea	Dizziness, vertigo, headache
Mirtazapine	Antidepressant	7.5–45 mg QD	Dyspepsia, nausea, increases appetite	Weight gain, drowsiness, dry mouth
Olanzapine	Atypical antipsychotic	2.5–10 mg QD	Dyspepsia, nausea, increases appetite	Weight gain, dyslipidemia, hyperglycemia, extrapyramidal symptoms, QT prolongation, sedation
SNRI *Duloxetine* *Venlafaxine*	Serotonin and norepinephrine reuptake inhibitors	*Duloxetine* 30–90 mg QD *Venlafaxine* 75- 225 mg QD	Constipation, abdominal pain	Abdominal pain, decreased appetite, nausea, drowsiness
SSRI *Citalopram* *Escitalopram* *Fluoxetine* *Paroxetine* *Sertraline*	Selective serotonin reuptake inhibitors	*Citalopram* 10–40 mg QD *Escitalopram* 5–20 mg QD *Fluoxetine* 10–40 mg QD *Paroxetine* 10–40 mg QD *Sertraline *25–150 mg QD	Globus, dysphagia constipation, bloating	Nausea, diarrhea (less common with paroxetine), decreased libido
Trazodone	Antidepressant, serotonin reuptake inhibitor/agonist	75–150 mg QD	Globus, dysphagia, functional chest pain, insomnia	Nausea, dizziness, drowsiness, xerostomia, blurry vision
Tricyclic Antidepressants A*mitriptyline,* *Desipramine, Imipramine, Nortriptyline*	Tricyclic antidepressant	25–150 mg QD	Abdominal pain, dyspepsia, nausea	Drowsiness, dry mouth constipation, QT prolongation
Quetiapine	Atypical antipsychotic	25–200 qd	Abdominal pain	Weight gain, dyslipidemia, sedation

### DGBI and ED intersection

#### DGBI in EDs

Patients with EDs frequently present with GI complaints, with patients with avoidant/restrictive food intake disorder (ARFID) showing particularly disproportionate GI symptom burden [13, 14]. In inpatient and outpatient ED settings, DGBI rates range from 39% and up to 98% [4, 15]. EDs may induce DGBI—while retrospectively reported, one study in irritable bowel syndrome (IBS) found that ED symptoms preceded IBS diagnosis for the majority (87%) of patients [16]. While ED sequalae (e.g. low weight in anorexia nervosa (AN)) may result in the pathologic processes leading to DGBI symptoms [17], the exact mechanisms involved in these pathways are not entirely clear. EDs have been shown to cause changes in the enteric, central, and autonomic nervous systems [3, 18–20], and several studies have noted that the dysmotility associated with DGBI (e.g. delayed emptying of the stomach, delayed colonic transit time) improves with refeeding [3]. However, other research has shown no association between DGBI and weight status (such as by body mass index (BMI)) [4]. Moreover, features of DGBI, such as bloating and distension, are present in equal frequencies across disparate ED phenotypes [21, 22]. This suggests DGBI in EDs may arise from a distinct mechanism rather than specific eating behaviors (e.g. self-induced vomiting) and physiological effects (e.g. low weight status) alone.

#### EDs in DGBI

Conversely, GI symptoms and their management can lead to disordered eating. Patients with DGBI commonly associate their symptoms with food [23], and may develop dysfunctional illness beliefs regarding their disease and eating-related consequences [24]. Others have hypothesized pathways through which EDs may manifest in the context of DBGI. For example, abdominal distension, a common symptom in DGBI, may increase body dysmorphia and lead to disordered eating practices. DGBI treatments, such as exclusion diets (e.g. low FODMAP diet, gluten-free diet), may also put patients at risk for, exacerbate, or cause patients with EDs in remission to relapse. One retrospective study found that that patients who had a history of trying an exclusion diet were over three times as likely to have avoidant/restrictive food intake disorder symptoms [25]. Another study also found that patients with IBS who screened positively for EDs were more adherent to the low FODMAP diet [26].

With growing awareness of this relationship, research has increasingly focused on identifying DGBIs among patients with EDs, with recent systematic reviews highlighting the most common symptoms [19, 27]. More research is certainly needed on shared (e.g., genetic) risk factors for ED and DGBI development. However, regardless of cause, understanding and managing DGBI symptoms is an essential component of care for patients with EDs, as untreated DGBIs may divert attention from ED pathology, perpetuate disordered eating behaviors, and impede nutritional rehabilitation [28]. In the sections below, we provide a summary of DGBI symptoms by anatomic region most frequently known to present in patients with EDs and their management—including esophageal symptoms (globus and functional dysphagia), gastroduodenal symptoms (functional dyspepsia and nausea), and bowel symptoms (abdominal pain, bloating and constipation).

## Esophageal symptoms: globus and functional dysphagia

### Esophageal physiology

In normal physiology, swallowing first consists of an oral preparatory phase in which food is chewed and consolidated into a formed mass (a bolus) under voluntary control mediated by nerves in the mouth and throat. In the pharyngeal (i.e. throat) phase, the bolus advances through the pharynx and into the esophagus, while avoiding the airway [29]. In the esophageal phase, peristalsis propels the bolus into the stomach, via skeletal muscle contractions in the first 1/3 of the esophagus and smooth muscle contractions in the lower 2/3 of the esophagus.

### Symptom definition

Esophageal DGBI common in EDs include globus and functional dysphagia [19, 27].

Globus is the non-painful sensation of a lump or a foreign body in the throat [30]. Patients may describe tightness, itching, tickling, mucus accumulation, or choking. However, globus does not impede passage of food, and in most patients, the sensation generally improves with swallowing [30].

In contrast, dysphagia is a subjective sensation of difficulty swallowing—that is, not just the sensation of something being in the throat, but that movement of food is impaired in the throat and esophagus. Dysphagia is further characterized by the location of the delay—if the symptoms occur when a swallow is started (oropharyngeal dysphagia) or when food is passing from the mouth to the stomach (esophageal dysphagia) [31]. Patients with oropharyngeal dysphagia may have coughing, choking, or regurgitation as they start to swallow, while patients with esophageal dysphagia may have the sensation that food and/or liquids are passing abnormally to the stomach *after* they start to swallow [31]. Odynophagia, defined as pain with swallowing, is rarely present in typical dysphagia or globus.

### Prevalence and pathophysiology in EDs

Both globus and dysphagia are frequently reported among patients with EDs, and may impede oral intake. In inpatients with EDs, globus and functional dysphagia have been reported in up to 5% and 16%, respectively [32].

### Clinical evaluation and management

#### Evaluation

While globus and dysphagia symptoms in EDs are often related to DGBI [22, 32], they require further evaluation to rule out structural or organic causes.

Barium contrast studies, in which a patient swallows a barium-containing solid or liquid which can be seen on x-ray, can be used to rule-out an obstruction/blockage in the throat or esophagus. Patients with EDs and dysphagia warrant GI referral for endoscopy to exclude structural abnormalities such as cancer or stricture (i.e. abnormal narrowing or tightening of the esophagus), or to assess the esophageal lining (mucosa) for signs of gastroesophageal reflux disease (GERD), or an allergic process (e.g. eosinophilic esophagitis). This is particularly important for patients with a history of vomiting (e.g. in bulimia nervosa) given the potential for the esophageal lining to be damaged by repeated exposure to acid from the stomach which may increase the risk of peptic complications, such as erosive esophagitis or Barrett’s esophagus [33, 34]—though this is not borne out in larger series [35].

Esophageal manometry may also be helpful to rule out major problems with the contraction/relaxation of muscles in the esophagus (i.e. motility disorder) causing dysphagia. Despite the relatively high dysphagia symptom prevalence in EDs (up to 16%) [32], there is limited evidence for objective esophageal motility disorders. A study of esophageal manometry in 23 inpatients with AN with symptoms of dysphagia, heartburn, or regurgitation showed that all patients had normal esophageal motility patterns but one patient, who had esophageal spasm [36]. With the exception of achalasia (a neuropathic disease characterized by impaired esophageal motility and relaxation of the lower esophageal sphincter), most esophageal motility abnormalities detected on manometry are treated based on symptoms (not based on the motility pattern).

#### Management

**Fig. 1:**
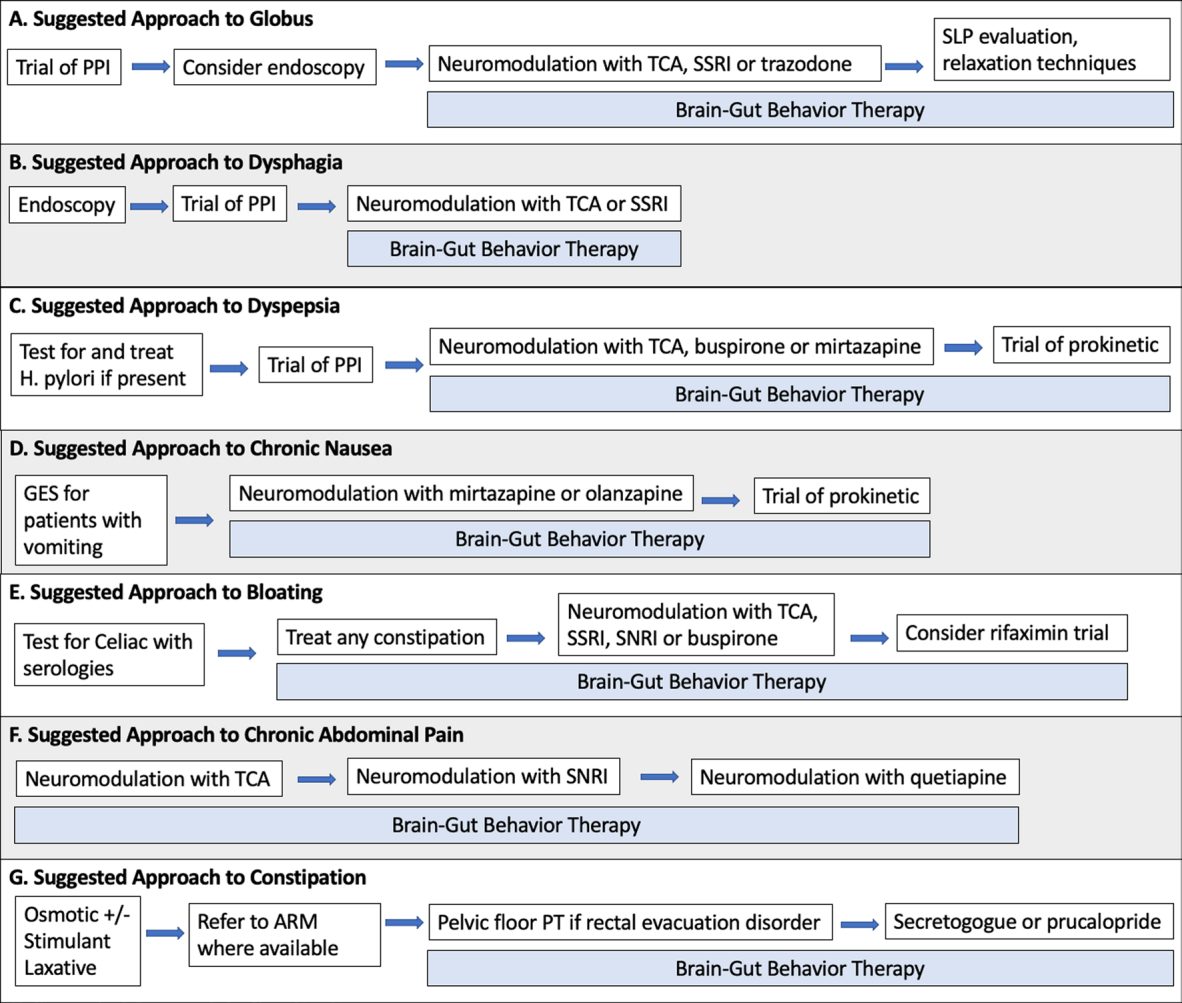
Suggested management for DGBI symptoms. PPI = proton pump inhibitor, TCA = tricyclic antidepressant, SSRI = selective serotonin reuptake inhibitor, SLP = speech language pathology, GES = gastric emptying scan, SNRI = serotonin norepinephrine reuptake inhibitor, ARM = anorectal manometry, PT = physical therapy

After excluding structural, mucosal, and motor disorders, the treatment of globus and functional dysphagia in patients with EDs involves reassurance, use of neuromodulators to treat visceral hypersensitivity, and complementary therapies (Fig. 1) [30, 37]. Explanation and reassurance are a mainstay of treatment, as both globus and dysphagia may improve over time without intervention. In a study of inpatients with EDs on admission for inpatient treatment, 19% had functional dysphagia, which had resolved in more than 80% by 12-month follow-up [22].

As many patients attribute their globus/dysphagia symptoms to gastroesophageal reflux disease (GERD), an initial step in management is often an empiric trial of acid-suppressant medication. In this situation, the medication is trialed for a set period of time (e.g. eight weeks), and if symptoms improve this is then indicative of GERD presence. Proton-pump inhibitors (PPIs) are recommended for these initial trials, as they are the most effective medical treatment, and should be taken once daily, 30 min before eating [38]. While GERD is highly prevalent in EDs [39], it is less likely to cause symptoms of globus or dysphagia in isolation.

For patients with normal workup and ongoing refractory symptoms, neuromodulators and brain-gut behavioral therapies may be helpful. Amitriptyline and paroxetine have both been studied in the treatment of functional esophageal symptoms, most recently in a randomized controlled trial of a general adult population with refractory globus which found that paroxetine was more effective than amitriptyline or lansoprazole [40]. Trazodone is another neuromodulator which may be used for functional esophageal symptoms [8]. Brain-gut behavior therapies (e.g. GI-focused exposure therapy, clinical hypnosis) are less studied for functional esophageal symptoms compared to other DGBIs, but one case series showed used of clinical hypnosis techniques reduced globus symptoms [41].

Speech and language pathology consultation may be considered as part of globus or dysphagia management, especially for hospitalized patients and those with a low BMI. A retrospective review of inpatients with severe AN found that symptoms of oropharyngeal dysphagia were present in 20% of inpatients, and 8% of the patients had abnormal swallow function testing requiring therapy that addressed the abnormalities (e.g. neuromuscular electrical stimulation) [42]. Neck and shoulder exercises and pharyngolaryngeal relaxation techniques administered by speech language pathologists have been shown to be helpful in globus sensation relief in some uncontrolled studies [30, 43]. Xerostomia or dry mouth, which is seen in increased frequency in patients with EDs, can mimic or worsen symptoms and should be treated with appropriate oral hygiene care [44]. Patients with functional dysphagia benefit from supportive guidelines about eating, including sitting upright and chewing well [37].

## Gastroduodenal symptoms: dyspepsia and nausea

### Gastric physiology

The stomach has an essential role in digestion, as the site for processing food and regulating its flow into the small intestine. The proximal stomach, where food first enters, is composed of the fundus and proximal corpus (or body), and serves as a temporary reservoir. In a process known as “accommodation,” the proximal stomach expands in response to a food bolus. This prevents an increase in gastric pressure with corresponding discomfort. Gastric contents are subsequently moved to the lower portion of the stomach (antrum), where food is churned and mixed into an appropriate size and consistency for further digestion. This digested food, “chyme”, is then passed through the pylorus which regulates emptying of the stomach contents into the small intestine (duodenum) [45]. Coordination of stomach motility requires complex interaction between the enteric nervous system, autonomic nervous system, and hormonal influences [45].

### Symptom definition

Gastroduodenal DGBIs common in EDs include functional dyspepsia and nausea [27].

Dyspepsia is a complex of symptoms including epigastric (i.e. upper stomach) pain or burning, early satiation (i.e. feeling very full shortly after eating a small portion), and post-meal discomfort, often attributed to the stomach and small intestine. When dyspepsia symptoms occur in the absence of underlying structural abnormalities, this is termed functional dyspepsia [46]. Nausea may occur with dyspepsia or in isolation.

### Prevalence and pathophysiology in EDs

Dyspepsia and nausea are some of the most commonly encountered symptoms among patients with EDs and may contribute to reduced intake [32]. Post-meal discomfort has been reported in up to over 90% of ED patients [47], and nausea was reported in 21% of ED outpatients [15]. Dyspepsia symptoms also have high overlap with ARFID symptoms (in up to 40% of outpatients) [13]. These symptoms are often attributed to ED-related alterations in gastric emptying and accommodation.

Gastric emptying is the most robustly studied objective GI measurement in both the general population and patients with EDs [4, 27] but likely has an outsized influence on our understanding of the stomach as it is measurable clinically using gastric emptying scans. In this test, the patient consumes radio-labeled food items, which allows specialized imaging to track the amount of time it takes the stomach to empty (i.e. gastric emptying time). Some early studies in patients with AN showed delayed gastric emptying (also known as gastroparesis) [33], which has been hypothesized to be related to the consequences of malnutrition–including smooth muscle breakdown, metabolic and hormonal imbalances. The relationship between weight rehabilitation and normalization of gastric emptying has varied among studies, with some showing improvement in gastric emptying times with improved nutrition, but not consistently [19, 22]. Gastric accommodation can be assessed using various imaging or pressure-sensor techniques in research, but not clinical, settings [48]. One study of adolescent female inpatients with AN showed decreased diameter of the antrum after eating, indicating that impaired accommodation could be a factor leading to dyspepsia for these patients [49]. Abnormal accommodation has also been invoked in patients with bulimia nervosa, although studies have had conflicting results [48].Gastric emptying and accommodation may be affected by consumption of large volumes of food ingested over a short period of time; this may explain why functional dyspepsia is frequently present in patients with binge-eating disorders [50].

### Clinical evaluation and management

#### Evaluation

The yield of investigations in patients with dyspepsia and/or nausea, including routine imaging or laboratory testing is low. Lab testing and/or imaging is warranted when patients with epigastric pain suggestive of a pancreatic or biliary source (e.g. pain radiating to the back) as both starvation-induced pancreatitis and refeeding pancreatitis are rare causes of abdominal pain in ED patients [51]. Imaging may also be considered to assess for compression of the duodenum between the aorta and superior mesenteric artery (SMA syndrome) in patients who have persistent refractory vomiting in the setting of significant weight loss.

Although a negative endoscopy is technically required to confirm a diagnosis of functional dyspepsia, dyspepsia guidelines from the American College of Gastroenterology and the Canadian Association of Gastroenterology discourage use of endoscopy in patients < 60 years of age without alarm symptoms (i.e. bleeding, anemia) [52]. Guidelines do suggest testing for H. pylori with stool antigen or breath testing. If positive, eradication of H. pylori should be pursued as test and treat strategy has been shown to be both cost-effective and improved dyspepsia symptoms versus placebo in two randomized controlled trials [52].

Tests of gastric physiology are not needed in most cases—as discussed above, measures of gastric accommodation are not routinely used in clinical settings, and the utility of gastric emptying scans has been questioned in recent years due to its non-reliable association with symptom severity and minimal evidence supporting therapies to improve delayed emptying [46]. However, gastric emptying scintigraphy may be indicated in some cases where refractory nausea and vomiting are present.

#### Management

Dyspepsia and nausea may improve with ED treatment, although in a long-term timeframe, supported by evidence in AN inpatients showing improvements with longer duration of nutritional rehabilitation and psychotherapy versus short-term refeeding [53].

A trial of empiric acid suppression with once-daily proton-pump inhibitor (PPI) is recommended as first line therapy for patients with dyspepsia after H. pylori infection has been excluded (Fig. 1) [54]. PPIs may also contribute to symptom improvement through anti-inflammatory effects in the small intestine, as impaired barrier function in the small intestinal lining has been proposed as a hypothetical cause of dyspepsia [55].

Tricyclic antidepressants (TCAs) are the most extensively studied neuromodulator for dyspepsia symptoms, and have shown some benefit, particularly for prominent abdominal pain [46, 52, 55]. Mirtazapine, a tetracyclic antidepressant, has been shown to improve functional dyspepsia and nausea symptoms and may be particularly useful in patients with EDs as it promotes weight gain, although may worsen constipation [55]. Buspirone, a serotonin-1A agonist has been found to improve accommodation by relaxing the proximal stomach and may be effective, particularly for post-meal discomfort [46]. Olanzapine, an atypical antipsychotic with serotonergic, dopaminergic and histaminergic effects may be used for patients with predominant symptoms of nausea [56]. In placebo-controlled trials with patients with AN, olanzapine has been shown to increase weight [57] and decrease obsessional thoughts [58]. Ondansetron, a centrally acting antiemetic, is commonly used for acute, self-limited nausea but has not been studied in chronic symptoms as seen in DGBIs.

Prokinetic agents have been used in patients with presumed or confirmed gastroparesis, but have several potential side effects, which may impede their use in patients with EDs. Metoclopramide is the most widely studied prokinetic, but carries a risk, albeit small, of neurologic symptoms (including irreversible tardive dyskinesia). Domperidone is not approved by the Food and Drug Administration in the United States due to case reports of ventricular arrhythmia and sudden cardiac death, however is widely used in Canada and Europe [59]. Erythromycin and azithromycin may be effective for short-term treatments because of the development of tachyphylaxis [60]. Prucalopride, a serotonin-4 agonist, is a prokinetic agent with effects on colonic transit time (discussed below) and gastric emptying [61] and may be beneficial for patients with delayed gastric emptying and constipation.

Brain-gut behavior therapies may also be helpful for functional dyspepsia and nausea symptoms. While less studied than in IBS, there is increasing research showing promise for GI-focused clinical hypnosis [62], exposure therapy [63], and multi-component therapies (e.g. cognitive techniques, relaxation training) [64].

## Bowel symptoms: constipation, abdominal pain and bloating

### Bowel physiology

In normal physiology, intestinal contents are propelled through the colon via contractions modulated through a network of nerves present in the wall of the GI tract [65]. When contents reach the rectum, they are evacuated as stool though a coordinated mechanism involving the abdominal, pelvic floor, anal, and rectal muscles [66].

### Symptom definition

Common bowel-related symptoms in EDs are constipation, abdominal pain, and bloating [27].

Constipation describes symptoms relating to defecation, including infrequent bowel movements (< 3 times per week), hard stools, straining, sensation of incomplete evacuation or needing to use manual maneuvers (e.g. digital disimpaction) to facilitate passage of stool. When these symptoms occur frequently and chronically without a clear etiology, this is defined as functional constipation, (sometimes referred to as chronic idiopathic constipation) [67]. Constipation is traditionally characterized based on three potential etiologies: slow-transit, disorders of rectal evacuation, and normal transit, but there is significant overlap between these processes [66]. In slow transit constipation, the transit time for contents to pass from beginning of the colon to the rectum is prolonged due to an incompletely understood process involving nerves, muscles, and mucosa. In disorders of rectal evacuation, transit time may be normal, but discoordination between pressure in the rectum and relaxation in the anus (“dyssynergic defecation”) prevents the passage of stool. Finally, in a third category of patients, transit time and stool burden are normal; the constipation symptoms of straining or bloating are driven by a presumed sensory dysfunction.

IBS is another DGBI characterized by abdominal pain which is related to defecation and associated with change in stool frequency or form. IBS subtypes—IBS with diarrhea (IBS-D), IBS with constipation (IBS-C), and IBS with mixed bowel habits (IBS-M)—are categorized based on the patients’ predominant stool consistency on days with abnormal stools. IBS and functional constipation fall along a continuum of abdominal pain and stool consistency [68].

Abdominal bloating is the subjective sensation of trapped gas or a feeling of fullness or pressure in the abdomen. Abdominal distension is an objective physical manifestation of an increase in abdominal girth [69]. Abdominal bloating and distension are frequently associated with IBS and functional constipation (as well as functional dyspepsia) but may also exist in isolation. Functional abdominal bloating and distension is a DGBI diagnosed in patients who have bloating and distension as their prominent GI symptom without a clear other cause [69].

### Prevalence and pathophysiology in EDs

Constipation-predominant DBGI is the most common DGBI among inpatients getting ED treatment [70], with functional bowel disorders reported in up to 98% of inpatients with EDs [32]. These disorders are equally present in the outpatient setting, where studies have shown that DGBI symptom severity—including constipation symptoms [71] and IBS symptoms [72]—was associated with greater ED pathology. In one study of 85 outpatients with AN, 93% of patients reported symptoms related to bowel-related DGBI, and prevalence increased with both lower BMI and duration of illness for longer than 5 years. Abdominal distension is one of the most commonly reported symptoms of inpatients with EDs, in up to 90% of patients in one study [73]. Abdominal distension and body dysmorphia have bidirectional relationship – distension may contribute to body dysmorphia and body dysmorphia may confound self-report of abdominal distension.

In patients with EDs, evidence for pathophysiologic underpinnings of constipation is varied. Early studies showed prolonged colonic transit time in patients with AN which normalized with nutritional rehabilitation, suggesting that malnutrition with associated smooth muscle breakdown and electrolyte disturbances may cause delayed transit times [74, 75]. However, other studies have shown no relationship between delayed transit time and BMI [76]. Disorders of rectal evacuation may occur in patients with EDs due to pelvic floor dysfunction in the setting of hypoestrogenism, low-protein diets, excessive exercise, or protracted evacuation efforts [20, 77]. Chiaroni et al. studied inpatients with AN and found that 42% had pelvic floor dysfunction which did not improve following 4-week refeeding period [74], while a more recent study of outpatients with chronic constipation did not show significant differences in pelvic floor and transit parameters between constipated patients with and without ED pathology [71].

Abdominal bloating has not been studied specifically in EDs, but studies of patients with functional bloating are likely relevant to patients with EDs. While most patients feel that their bloating is caused by increased gas and/or stool which is produced or “trapped” in their intestines, imaging performed during bloating episodes has shown largely normal gas and stool volume [78, 79]. Instead, increased *perception* of gas, via visceral hypersensitivity, rather than an excess of gas and/or stool, is a key driver of symptoms. As an example, patients with functional bloating have been shown to develop symptoms in response to normal gas volumes which are tolerated by healthy controls [78]. An underrecognized cause of abdominal distention is an abnormal viscero-somatic reflex (also known as abdomino-phrenic reflex), which may represent the primary cause of distention in those without severe gastrointestinal motility disorders— distention of the stomach from eating results in an inappropriate downward (instead of normal upward) contraction of the diaphragm, pushing abdominal contents downward. Paired with inappropriate relaxation of the abdominal wall muscles, normal-volume abdominal content is pushed outward, resulting in visible distension out of proportion to the contents ingested [78].

### Clinical evaluation and management

#### Evaluation

The yield of laboratory evaluation of constipation and/or bloating is low, but can be considered to rule out hypothyroidism, and celiac disease in patients with other clinical features of these processes [67]. For patients with bloating, breath testing for carbohydrate malabsorption or small intestinal bacterial overgrowth may be helpful, but there are significant limitations in standardization and interpretation (Fig. 1) [69].

Motility testing for bowel-related symptoms is rarely indicated initially, and there is no clinically available test to diagnose an abnormal viscero-somatic reflex. Anorectal manometry can be helpful for patients with symptoms of constipation who are not responding to initial pharmacologic therapy and can guide selection of candidates for pelvic floor physical therapy/biofeedback therapy (described below). A more intensive workup may be considered for patients with neurologic symptoms, autonomic dysfunction, joint hypermobility or significant, objective distension, however it should be noted that most symptom management is not contingent on underlying diagnoses.

#### Management

In general, reassurance and explanation of normal and pathophysiology of bowel symptoms can be therapeutic in and of itself. For example, providers can describe the normal range of bowel movement frequency and consistency for patients who perceive they are constipated or explain abnormal viscero-somatic reflex in bloating. Patients’ medications should be reviewed to assess for medications which may be contributing to constipation, such as anticholinergic agents, tricyclic antidepressants, and iron supplements.

Osmotic laxatives (e.g. polyethylene glycol) are first-line agents in managing constipation and are generally well-tolerated, although can cause dose-dependent bloating and gas. Stimulant laxatives (e.g. bisacodyl, senna) are also available over the counter and frequently used, although have high potential for abuse as they produce a more high-volume stool output [80]. A commonly held misconception is that long term use of stimulant laxatives can lead to colonic nerve damage; this has been disproven in well-designed trials [81]. However, the abuse potential of stimulant laxatives is problematic as patients, and particularly those with EDs, may become accustomed to the cathartic feeling of a large, loose bowel movement—and perceive they are constipated without this. When abused, stimulant laxatives may result in electrolyte disturbances and kidney dysfunction [80].

Secretagogues (e.g. linaclotide, plecanatide, lubiprostone, tenapanor) are prescription medications which have been studied extensively in functional constipation and IBS-C. In addition to improving stool frequency and consistency, secretagogues may also improve abdominal pain due to attenuation of GI tract pain signaling [67], and have been shown to reduce bloating in clinical trials with IBS-C [82]. While abuse of secretagogues has not been described in the literature, it is certainly possible that individuals determined to purge can exploit these medications, particularly linaclotide as it is most likely to cause diarrhea (Table 3).

Prucalopride– a 5HT-4 receptor agonist—has prokinetic effects throughout the GI tract, and may be useful for patients with concomitant upper GI symptoms (e.g. nausea, dyspepsia). Prucalopride may also reduce bloating in patients with chronic constipation and gastroparesis through its prokinetic effects, but it is unclear if the reduction in bloating is due to the medication alone or due to its effect in promoting bowel movements [78, 83].

Neuromodulators, specifically amitriptyline, escitalopram, and buspirone have been shown to improve IBS symptoms including bloating, although no study to date has focused on bloating as a single symptom [69]. Anecdotally, we frequently use the serotonin and norepinephrine reuptake inhibitor (SNRI) duloxetine in patients with bloating and constipation not responding to normalization of bowel habits. For patients with abdominal pain-predominant symptoms, TCAs are first line therapy, with secondary amines (e.g. desipramine and imipramine) favored due fewer anticholinergic and antihistaminic side effects [8]. SNRIs are also used for pain-predominant symptoms [8].

Brain-gut behavior therapy has been most extensively studied in IBS, with both GI-focused cognitive behavioral therapies and clinical hypnosis tested in over 30 randomized controlled trials [84]. Brain-gut behavior therapy is recommended as part of comprehensive management of pain in DGBI [85], and may be helpful for bloating associated with IBS [69].

Pelvic floor biofeedback therapy may be used in patients with signs of rectal evacuation disorder on anorectal manometry [86], and may alleviate abdominal pain and bloating in addition to infrequent and hard stools [87].

Dietary approaches are often used in management of IBS and bloating. However, these diets can be restrictive and likely not appropriate for patients with pre-existing EDs since they could exacerbate underlying restrictive tendencies or reactivate maladaptive cognitions. Agents which modulate the microbiome have also been explored as treatments, although studies for pre- and probiotics have had contradictory results [83]. Rifaximin, a minimally absorbed broad spectrum antibiotic has been shown to have some effect on bloating in patients with IBS-D [69]. However, its utility may be limited by symptom recurrence after the initial two week course and need for re-treatment [83]. Evidence for other therapies, such as biofeedback for an abnormal viscerosomatic reflex, is still emerging.

## Conclusion

DGBI and EDs have a complex, bidirectional relationship. Symptoms of DGBI, such as globus, dyspepsia, nausea, bloating, abdominal pain, and constipation are frequently present in patients with EDs and may precede, augment, or perpetuate disordered eating. Understanding the pathophysiology and DGBI diagnoses related to these common symptoms is critical for the care of ED patients. Reassurance and explanation of DGBI symptoms form the cornerstone of their management and can be essential to building a therapeutic alliance. Pharmacological approaches, via prokinetic and neuromodulatory agents, are effective in DGBI management, and some may have a dual purpose in treatment of EDs. Overall, we propose that acknowledgment and thoughtful management of DGBI symptoms will advance the care of patients with EDs.

## Data Availability

Not applicable.

## Abbreviations

DGBI: Disorder of gut–brain interaction
ED: Eating disorder
GI: Gastrointestinal
TCA: Tricyclic antidepressants
SSRI: Selective serotonin reuptake inhibitor
GERD: Gastroesophageal reflux disease
AN: Anorexia nervosa
BN: Bulimia nervosa
FODMAP: Fermentable oligosaccharides, disaccharides, monosaccharides and polyols
BMI: Body mass index
PPI: Proton pump inhibitor
IBS: Irritable bowel syndrome
SNRI: Serotonin norepinephrine reuptake inhibitor
